# Auxins and grass shoot architecture: how the most important hormone makes the most important plants

**DOI:** 10.1093/jxb/erad288

**Published:** 2023-07-20

**Authors:** Alex Wakeman, Tom Bennett

**Affiliations:** School of Biology, Faculty of Biological Sciences, University of Leeds, Leeds LS2 9JT, UK; School of Biology, Faculty of Biological Sciences, University of Leeds, Leeds LS2 9JT, UK; Duke University, USA

**Keywords:** Auxin, cereal, grain, grass, inflorescence, *Poaceae*, shoot architecture, shoot development, tillering

## Abstract

Cereals are a group of grasses cultivated by humans for their grain. It is from these cereal grains that the majority of all calories consumed by humans are derived. The production of these grains is the result of the development of a series of hierarchical reproductive structures that form the distinct shoot architecture of the grasses. Being spatiotemporally complex, the coordination of grass shoot development is tightly controlled by a network of genes and signals, including the key phytohormone auxin. Hormonal manipulation has therefore been identified as a promising potential approach to increasing cereal crop yields and therefore ultimately global food security. Recent work translating the substantial body of auxin research from model plants into cereal crop species is revealing the contribution of auxin biosynthesis, transport, and signalling to the development of grass shoot architecture. This review discusses this still-maturing knowledge base and examines the possibility that changes in auxin biology could have been a causative agent in the evolution of differences in shoot architecture between key grass species, or could underpin the future selective breeding of cereal crops.

## Teaser

We review recent advances in understanding auxin biology in the grass family, and explore the possibility that changes in auxin biology led to differences in shoot architecture between grass species.

## Introduction

The grass family (*Poaceae*) of flowering plants includes key species grown, cultivated, and bred by humans for their grain—the cereal crops. The high yields produced by these cereals have underpinned the development and maintenance of all agrarian societies since the Neolithic revolution, and continue to be vital to global food security. Between them, the ‘trinity’ of wheat, rice, and maize are predicted to account for more than half of all human calorie consumption, with the remaining cereal crops, such as barley, millets, sorghum, rye, and oats, also contributing significantly to modern global diets (Cassman, 1999; Tilman *et al.*, 2011). In the face of increasing global population and climate change-driven loss of arable land, the development of new cereal breeds that can produce greater yields has been identified as an essential, perhaps ‘the’ essential, challenge of modern plant science.

**Fig. 1.**
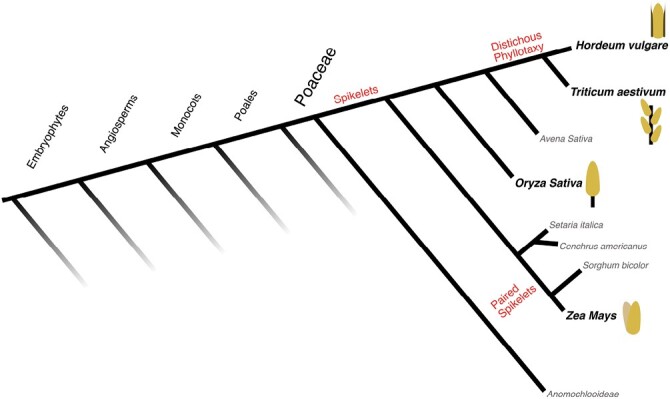
Evolutionary comparison of the *Poaceae*. Species discussed in this review are in bold and accompanied by an inflorescence (spikelet) diagram, in which each floret is represented by a yellow oval, *Hordeum vulgare* (barley) (with infertile lateral spikelets shown in black), *Triticum aestivum* (wheat), *Oryza sativa* (rice), and *Zea mays* (maize). Other cereals that are not extensively studied or discussed in this review are included in grey. Defining features of inflorescence development are labelled in red.

**Fig. 2.**
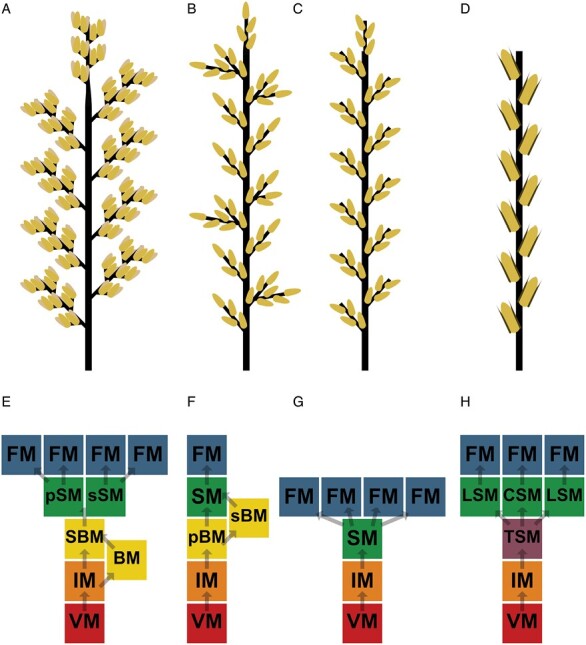
Diagrams of the shoot architecture of the main *Poaceae* species discussed in this review. Each diagram represents an entire spike in black, and each yellow oval represents a floret, each of which can give rise to a single grain. (A) Maize tassel (male inflorescence): produces pairs of spikelets on short branches along its lateral branches and main spike; each spikelet then produces two florets. (B) Rice panicle: produces multiple orders of tillers; also, shown here with each spikelet producing a single fertile floret; significant support exists for a ‘three-floret spikelet’ hypothesis that postulates that two sterile lemmas on each inflorescence are in fact lateral florets. These florets are not included here due to their still hypothetical nature (Ren *et al.*, 2020). (C) Wheat ear: produces mulitfloreted spikelets. (D) Barley ear (two-rowed): produces a unifloreted central spikelet, flanked by two lateral spikelets, which are typically sterile and represented by thin dark ovals. (E–H) Diagrams of shoot meristem development in the main *Poaceae* species discussed herein. (E) Maize tassel (male inflorescence). VM, vegetative meristem; IM, inflorescence meristem; BM, branch meristem; SBM, short branch meristem; pSM, pedicellate spikelet meristem; sSM, sessile spikelet meristem; FM, floret meristem. (F) Rice panicle. pBM, primary branch meristem; sBM, secondary branch meristem; SM: spikelet meristem. (G) Wheat ear. (H) Barley ear. TSM, triple spikelet meristem; CSM, central spikelet meristem; LSM, lateral spikelet meristem.

The *Poaceae* probably first emerged as a distinct family at the start of the Cretaceous period, with recent estimates placing their diversification from other *Poales* typically ~80–100 million years ago (MYA) and as far back as 130 MYA (Prasad *et al.*, 2005; Strömberg, 2011; Polissar *et al.*, 2019; Gallaher *et al.*, 2022; Peppe *et al.*, 2023). In terms of abundance and diversity, the *Poaceae* have become one of the most successful plant families, comprising an estimated 11 000 species (Bouchenak-Khelladi *et al*., 2014; Linder *et al.*, 2018) (Fig. 1). They are also the dominant organisms in a wide range of natural ecosystems (i.e. grasslands), the first of which are thought to have arisen in Africa ~21 MYA. Compared with other *Poales*, grasses have a series of distinctive traits, such as improved stomata, environmental tolerance, and flexible growth (Chen *et al.*, 2017; Linder *et al.*, 2018). Grasses also share a complex shoot architecture, which underlies their ability to rapidly colonize land, grow quickly, and produce large amounts of grain (Fig. 2). Grasses typically produce multiple ‘tillers’ (a grass-specific term for vegetative branches) during the vegetative phase, which allows them to rapidly expand their ground coverage, and take up and store nutrients. Furthermore, during the vegetative phase, grass shoot meristems remain at the base of the shoot, producing erect leaves, meaning that grazing by herbivores does not destroy meristems, only the leaf blades. While many monocots share this basic pattern of shoot development, it is probably their vigorous vegetative shoot growth that especially contributes to grasses dominating some ecosystems. After the transition to flowering, a proportion of the tillers will initiate primary inflorescences (often called spikes, ears, or panicles depending on the species), while other tillers undergo senescence and nutrient remobilization to flowering tillers. This flexible and reversible pattern of tiller initiation allows grasses to dominate light capture during the vegetative phase, without committing to maintaining every tiller. The primary inflorescences of grasses in turn bear secondary inflorescences typically referred to as ‘spikelets’. Each spikelet initiates a certain number of florets (depending on the species), each of which can become fertilized and produce a single seed (Fig. 2). The ultimate success of seed production is therefore the result of the sequential development of tillers, spikes, spikelets, and florets, which can vary greatly between grass species, and even between ecotypes/cultivars of the same species.

The development of these structures results from the action of increasingly specialized shoot meristems (Fig. 2). Leaves are initiated by the primary vegetative shoot apical meristem in the developing plant, and each leaf is associated with an axillary shoot meristem. Activation of these axillary meristems leads to the formation of a new shoot axis, a tiller. After floral transition, the shoot meristems in the primary shoot and major tillers will undergo conversion to form primary inflorescence meristems, which can be characterized as a type of reproductive shoot meristem, since they do not directly initiate to flowers (Schultz and Haughn, 1991; Tanaka *et al.*, 2013; Bommert and Whipple, 2018; Koppolu and Schnurbusch, 2019). The primary inflorescence meristem initiates a series of bracts (leaf-like organs) each of which is associated with an axillary meristem. These axillary meristems may be specified as branch meristems that give rise to primary inflorescence branches (which have the same developmental potential as the main inflorescence), or as spikelet meristems. Spikelet meristems are true inflorescence meristems that initiate glumes, and then lemmas (bract-like structures) along their length, each of which is associated with a floral meristem that forms a single floret. Many grass spikelets (e.g. barley and rice) only give rise to a single fertile floral meristem, but others may produce multiple florets per spikelet (e.g. wheat).

It is likely that the flexible pattern of vegetative and reproductive shoot architecture in grasses has contributed to the enormous success of the grass family, by allowing a diverse set of morphologies that can colonize different ecosystems and produce a large quantity of seed that is easily dispersed (Chen *et al.*, 2017; McSteen and Kellogg, 2022). Moreover, these shoot architectural features and the grains they produce have become key elements supporting modern and historical human societies. Thus, understanding the development of grass shoot architecture—and therefore how it can be improved to support future agricultural demands—is a question of some importance. Yield improvements through conventional breeding have already resulted in substantial changes to the shoot architecture of elite cereal varieties, whether through changes in tiller number, tiller angle, leaf size and shape, flowering time and synchronicity, inflorescence size and shape, increases in total grain mass, or by improvements to other reproductive traits, such as free-threshing and inflorescence harvestability (Li *et al.*, 2018; Sakuma and Schnurbusch, 2020; Liu *et al*., 2021). The transformation of the weed teosinte into the high-yielding modern maize crop represents the most dramatic example of these breeding-driven changes in shoot architecture (Q. Chen *et al*., 2020, 2021). A key goal for cereal research must therefore be to provide the deep biological understanding to allow more precise alteration of cereal shoot architecture, to deliver specific cereal ‘ideotypes’, with optimal morphology for particular purposes, in particular environments. Although shoot architecture is similar between cereals, what is required of these ideotypes varies greatly between species. For instance, increased panicle branching is important in rice, but irrelevant in wheat, where the production of a small number of highly fertile ears is a more pertinent aim (Miura *et al.*, 2010; Sreenivasulu and Schnurbusch, 2012; Wang *et al*., 2018;  Y. Chen *et al.*, 2020). Therefore, species-specific knowledge and a robust understanding of differences in shoot development between cereals is crucial in understanding which genes are most relevant to a particular shoot structure in a particular species.

The phytohormone auxin [indole-3-acetic acid (IAA)] has well-established, key roles in shoot meristem development (Pautler *et al.*, 2013; Wang *et al*., 2018), and in determining patterns of branching within shoot systems (Wang *et al*., 2018). As such, auxin might be expected to play a major role in the development of grass shoot architecture, a hypothesis largely borne out by work in grasses over the last decade (Gallavotti *et al.*, 2008; Phillips *et al.*, 2011; Matthes *et al.*, 2019; Y. Chen *et al.*, 2020; Dong *et al.*, 2021; J. Li *et al.*, 2021; Qiao *et al.*, 2021). Here, we aim to review recent advances in our understanding of auxin-driven shoot development in grasses. We have primarily focused on how auxin influences the number of organs produced in the shoot, through its effects on the number of different shoot meristems produced, and their relative activity. We acknowledge that there are many other important components of shoot architecture that are influenced by auxin, such as tiller angle, leaf shape, and flowering time (Xia *et al.*, 2012; H. Li *et al.*, 2021; Zhao *et al.*, 2023), but there was not space to cover all these aspects in this review.

The basic mechanisms of auxin biosynthesis (Gallavotti *et al.*, 2008; Phillips *et al.*, 2011), conjugation, degradation, signalling, and transport are generally highly conserved across the land plant group (Mano and Nemoto, 2012; Casanova-Sáez *et al*., 2021), and grasses are no exception to this, containing paralogues of all known auxin-related gene families (Table 1). Indeed, there is no reason to believe that these basic biochemical processes are fundamentally different in grasses from other plants. Furthermore, current evidence suggests that—again in fundamental aspects—grass development is regulated by auxin in the same way as other angiosperms. Auxin drives the formation of organs in the shoot meristems in grasses, and then regulates the activity of those meristems, and therefore the number and arrangement of mature organs formed (Kellogg, 2022). However, the devil is in the detail. Given the central importance of auxin in plant development, it is very plausible that changes in the timing, pattern, and level of expression of auxin-related genes, or changes in the specific activity of the encoded proteins might explain the differences in shoot development between different grass species, or between grasses and other flowering plants. This is the central hypothesis that this review aims to examine. We will focus on the three best characterized areas of auxin biology in grasses; TAA/YUC-mediated auxin synthesis, PIN-mediated auxin transport, and TIR1/AFB-mediated nuclear auxin signalling.

**Table 1.** Copy number of auxin-related genes in cereal species

Family	Species	Known homologues
**TAA/TAR**	Rice	4
	Maize	6
	Wheat	15
	Barley	1
**YUC**	Rice	14
	Maize	14
	Wheat	16
	Barley	3
**PIN**	Rice	12
	Maize	15
	Wheat	44
	Sorghum	11
**Aux/IAA**	Rice	31
	Maize	34
	Wheat	84
	Barley	36
**ARF**	Rice	25
	Maize	36
	Wheat	67
	Barley	25
**TIR1/AFB**	Rice	5
	Maize	6
	Wheat	11

TAAs: rice (T. Zhang *et al.*, 2018), maize (Chourey *et al*., 2010; Phillips *et al.*, 2011), wheat (Shao *et al.*, 2017), barley (Bai *et al.*, 2017).

YUCs: rice (T. Zhang *et al.*, 2018), maize (Gallavotti *et al.*, 2008; Chourey *et al*., 2010; Li *et al*., 2015), wheat (Li *et al.*, 2014; Yang *et al.*, 2021), barley (Mudgett and Zhao, 2022).

PINs (Bennett *et al*., 2014a): rice (Wang *et al.*, 2009; Miyashita *et al*., 2010), maize (Forestan *et al*., 2012; O’Connor *et al.*, 2014; Yue *et al.*, 2015), wheat (Kumar *et al.*, 2021), sorghum (Shen *et al.*, 2010).

AUX/IAAs: rice (Jain *et al.*, 2006), maize (Ludwig *et al*., 2013), wheat (Chaudhary *et al*., 2023), barley (Shi *et al.*, 2020).

ARFs: rice (Wang *et al.*, 2007), maize (Liu *et al.*, 2011; Wang *et al.*, 2012; Galli *et al.*, 2018), wheat (Qiao *et al.*, 2018; J. Li *et al.*, 2021; Chaudhary *et al*., 2023), sorghum (Wang *et al.*, 2010).

TIR1/AFBs: rice (Matthes *et al.*, 2019; Guo *et al.*, 2021), maize (Matthes *et al.*, 2019), wheat (Gidhi *et al*., 2023).

## TAA/YUC-mediated auxin biosynthesis

The core IAA biosynthesis pathway in plants involves a two-step reaction, beginning with the reversible conversion of tryptophan to indole-3-pyruvic acid (IPyA), catalysed by TRYPTOPHAN AMINOTRANSFERASE OF ARABIDOPSIS (TAA) family enzymes (Zhao, 2018; Casanova-Sáez and Voß, 2019). IPyA then undergoes oxidative decarboxylation, catalysed by YUCCA (YUC)-type monooxygenases, to yield IAA. Other IAA biosynthetic pathways exist in plants, and appear to be conserved in grasses, but are functionally less important than the TAA/YUC pathway (Mano and Nemoto, 2012; Korasick *et al*., 2013). Many different *TAA* and *YUC* paralogues exist in flowering plants, and the exact number of *TAA* and *YUC* genes varies between species (Table 1), as do the location, timing, and magnitude of expression of each gene (Poulet and Kriechbaumer, 2017). Phylogenetic studies have categorized *TAA* genes into two major clades, the first containing the key Arabidopsis genes *AtTAA1*, *AtTAR1* (*TAA-RELATED1*), and *AtTAR2*, and the second containing a group of alliinase-related *TAA* genes, including *AtTAR3* and *AtTAR4* which have been less studied (Chourey *et al*., 2010; Matthes *et al.*, 2019). The *YUC* phylogeny has four main branches. Two of these contain Arabidopsis shoot-expressed *YUC* genes and homologues (*AtYUC1*/*AtYUC4* in one clade and *AtYUC2*/*AtYUC6* in the other) (Cheng *et al*., 2006). A third clade consists of all Arabidopsis root-expressed genes (*AtYUC3*, *AtYUC5*, *AtYUC7*, *AtYUC8*, and *AtYUC9*) (Chen *et al.*, 2014), while the final clade consists of embryo-expressed *AtYUC10* and *AtYUC11* (Cheng *et al*., 2006).

The *TAA* and *YUC* genes identified in grasses allow us to conclude that, as in Arabidopsis, different *TAA* and *YUC* genes have specific roles within a given species. For instance, *ZmYUC1*, *OsYUC9*, and *OsYUC11* have important functions in controlling auxin concentration during embryo development (Abu-Zaitoon *et al.*, 2012; Bernardi *et al.*, 2012), an equivalent role to *AtYUC1*, *AtYUC4*, and *AtYUC10* which are essential in embryogenesis in Arabidopsis (Cheng *et al*., 2007). Intriguingly, several grass *TAA* and *YUC* genes appear to either differ in expression and function from their Arabidopsis paralogues or are absent in Arabidopsis entirely. For instance, maize and rice *YUC* genes *ZmYUC2*, *ZmYUC4*, and *OsYUC7* cluster with the Arabidopsis root-expressed *YUC* clade (*AtYUC3, AtYUC5*, *AtYUC7*, *AtYUC8*, and AtYUC9), but have been found to be highly expressed in the shoot (Chen *et al.*, 2014; Matthes *et al.*, 2019). Perhaps most intriguing are the maize and rice genes *ZmYUC7* (Gallavotti *et al.*, 2008) and *OsYUC8*, which cluster together, but have no obvious Arabidopsis paralogues, presenting the possibility of a monocot-specific subclade of *YUC* genes (Fujino *et al.*, 2008; Qin *et al.*, 2017). The role of this clade in shoot development is unclear; no functional work has been published regarding ZmYUC7. Meanwhile, OsYUC8 has been implicated in leaf development, but its precise role is still unclear (Zhou *et al.*, 2023). Expression data do suggest that *ZmYUC7* is highly expressed in the seed, *OsYUC8* is highly expressed in the immature inflorescence, and both are highly expressed in the shoot and embryo, identifying these genes as relevant for further investigation.

In maize, *Sparse Inflorescence1* (*Spi1*) encodes a YUC protein with an essential role in inflorescence development (Gallavotti *et al.*, 2008). Unlike many *yuc* single mutants in dicots, the *spi1* single mutant showed extreme developmental effects, with reduced tillers, spikelets on the tassel (male inflorescence), tassel length, spikelet pair meristems on the ear (female inflorescence), ear length, and plant height, as well as producing spikelet meristems at atypical locations on developing male and female inflorescences and at a greatly reduced number (Barazesh *et al*., 2009). Other studies in maize found the mutant line *defective endosperm18* (*de18*) to produce smaller grain than the wild type, with a 40% reduction in endosperm dry mass (Bernardi *et al.*, 2019). The effect was found to be rescued by the application of either of the synthetic auxins NAA (1-naphthaleneacetic acid) (Torti *et al*., 1986) or 2,4-D (2,4-dichlorophenoxyacetic acid) (Lur and Setter, 1993). The *De18* locus was identified as being tightly linked with an allele of *ZmYUC1* which contained rearrangements and a premature stop codon, leading to a truncated YUC1 protein. The failure of the *de18* mutants to produce sufficient auxin in the endosperm thus appears to account for the observed change in grain development. In barley, an allele of *HvYUC4* was identified as the causative gene of the mutant line *male sterile genetic38* (*msg38*), which produces shrunken pollen grains but no other developmental defects, suggesting that auxin synthesis is essential for proper pollen grain development (Amanda *et al.*, 2022; Mudgett and Zhao, 2022). *HvYUC4* is closely related to *AtYUC2* and *AtYUC6*, and the disruption of either of these genes also results in male sterility in Arabidopsis. *HvYUC2* is categorized into the same clade as *HvYUC4*, but its knockout does not result in sterility and the barley *yuc2 yuc4* double mutants are indistinct from the *yuc4* single mutant. This suggests that *HvYUC2* is neo-functional relative to *HvYUC4*, although what its function might be is not currently clear. In rice, the closest paralogue of *HvYUC4* is *OsYUC4,* but it is overexpression, rather than mutation, of the gene that caused improper pollen development (Zhao *et al.*, 2013). While this example is perhaps only tangentially relevant to shoot architecture, it supports the idea that change in auxin synthesis gene expression could contribute to changes in shoot and reproductive architecture.

A maize paralogue of *TAR2* called *Vanishing Tassel2* (*VT2*) has also been identified through genetic screens. Much like the *spi1* mutants, *vt2* single mutant maize also exhibited significant defects in inflorescence development, producing fewer ears that were shorter and had fewer spikelets (Phillips *et al.*, 2011). Interestingly, *spi1 vt2* double mutants showed little difference from either of the single mutants, suggesting that these proteins function together in inflorescence-specific IAA biosynthesis. In rice, the *tillering and small grain1* (*tsg1*) mutant exhibits increased tillering, but decreased panicle and grain size and number, which was related to a decrease in endogenous auxin levels. *TSG1* was identified as an allele of the tryptophan aminotransferase gene *FISH BONE* (*FIB*) (Guo *et al.*, 2020)*. FISH BONE* had previously been identified as a paralogue of *AtTAR2* and its mutation was shown to result in disruption to panicle and flower development (Yoshikawa *et al.*, 2014). Interestingly, single mutants in other rice *TAA* paralogues, such as *OsTAR1*, show very little phenotypic difference from the wild type. This implies that *TSG1* has a major or more functionally distinct role from *OsTAR1* and others. In addition to the *TAR2* paralogues *ZmVT2* and *OsTSG1*, the closely related wheat gene *TaTAR2.1* has also been implicated in shoot development. *TaTAR2.1* knockdown lines showed a reduction in grain mass, grain number, spikelet number, and height (Shao *et al.*, 2017). The expression and functional data of these related genes in maize, rice, and wheat show they are similar but distinct in their influence on shoot development. *ZmVT2* appears to influence spikelet number, but *OsTSG1* does not. *TaTAR2.1* and *OsTSG1* both appear to affect grain development, but *ZmVT2* does not. *OsTSG1* and *OsTAR1* have been reported to be highly expressed in mature inflorescences, whereas *ZmVT2* instead is more highly expressed in immature inflorescences. *vt2* and *tar2.1* mutants both reduce tiller/spike production, while *tsg1* mutants increase tiller production. Thus, the limited functional data currently available support the idea that changes in the timing/location of auxin biosynthesis might underpin some of the differences in shoot architecture between grass species.

Functional evidence for specific effects of *TAA* and *YUC* activity on grass shoot architecture is still sparse, though the examples discussed here show their involvement in these developmental processes. The differences in closely related *TAA* and *YUC* genes in a variety of cereals show that the role of auxin synthesis in cereal tillering is complex and species dependent. Although this does not equate to direct evidence that innovation in biosynthesis resulted in developmental innovation, it does support the theory that this could have occurred, and the patterns of phylogenetic conservation and distinction amongst grasses and between grasses and other plant species further supports this possibility.

## PIN-mediated auxin transport

As a huge number of studies have shown over the last two decades, a fundamental aspect of auxin-regulated plant development is the highly controlled distribution of auxin among tissues by specific auxin transport mechanisms (Křeček *et al.*, 2009; Bennett *et al*., 2014b; Bennett, 2015; Zhou and Luo, 2018). There are three groups of plasma membrane auxin transporters: the AUX1/LAX auxin influx carriers; the ATP-BINDING CASSETTE subfamily B (ABCB) efflux transporters (Yue *et al.*, 2015; Chai and Subudhi, 2016); and the PIN auxin efflux transporters. The PIN transporters are the most well studied, particularly because they show polar localization in many cells that is consistent with the observed directionality of auxin transport, because they show dynamic intracellular behaviour, and because they have well-developed imaging resources including functional green fluorescent protein (GFP)-tagged protein fusions and useful, highly specific antibodies. They are also most strongly associated with development, with many *pin* mutants showing specific developmental patterning defects that can be associated with specific changes in auxin transport (Paponov *et al.*, 2005; Forestan and Varotto, 2012; Wang *et al.*, 2022). AUX1/LAX and ABCBs are less well chracterized in cereals, and we thus focus on PIN-mediated auxin transport here, but, where available, studies do suggest that these transporters affect shoot development (Huang *et al.*, 2017; Zhu *et al.*, 2022).

PINs have been identified in all land plants (Adamowski and Friml, 2015; Zhou and Luo, 2018), and it has previously been proposed that polar auxin transport is one of the essential molecular innovations that resulted in the widespread adoption of embryophyte specific structures and processes that led to the success of the land plants (Bennett, 2015). In angiosperms, there are four canonical PIN clades (PIN1, PIN11, PIN3, and PIN2) which have long intracellular loop domains that function as regulatory modules, and which often have polar localizations, along with four clades of semi- (PIN6) or non-canonical clades (PIN5, PIN12, and PIN8) with divergent structural features (Bennett *et al.*, 2014a). These clades are well conserved across the angiosperms, including in monocots, and the broader Poales. However, there has been considerable change and innovation in the PIN family specifically in the *Poaceae* (Bennett *et al*., 2014a) (Table 1). For instance, there is a conserved triplication of the PIN5 clade in grasses, along with the apparent complete loss of the PIN6 clade. Proteins in the PIN3 clade in grasses are so divergent in sequence relative to other angiosperms that they were originally classified as a completely distinct grass-specific clade (PIN10). In addition, the PIN1 clade has undergone an apparent triplication in grasses, leading to two clades containing PIN1-like sequences (PIN1a and PIN1b) and a third containing a highly divergent non-canonical PIN protein, PIN9. This latter clade does seem to derive from a PIN1-like sequence but is sufficiently different to warrant a separate name. It has therefore been proposed that the grass-specific complement of PINs might be involved in grass-specific innovations in shoot architecture (Bennett *et al.*, 2014a).

In the case of *PIN1a* and *PIN1b* there is certainly evidence that they are functionally distinct (O’Connor *et al.*, 2014). Analysis of *PIN1a*, *PIN1b*, and *PIN11/SISTER OF PIN1* (*SoPIN1*) in the model grass species *Brachypodium distachyon* (Brachypodium) shows that these three genes have distinct expression domains in spike meristems that collectively resemble *PIN1* expression in Arabidopsis shoot meristems, with *SoPIN1* expressed in the epidermis, *PIN1b* in developing vascular strands, and *PIN1a* more broadly in the internal tissues (O’Connor *et al.*, 2014). SoPIN1 is needed for the formation of auxin maxima in the Brachypodium spike meristems, and *sopin1* mutants resemble classic *pin1* mutants in Arabidopsis. Since the *Brassicaceae* have lost the *PIN11* clade, it is assumed that Arabidopsis PIN1 is therefore functionally equivalent to both SoPIN1 and PIN1a/PIN1b from grasses. The distinction between the *PIN1a* and *PIN1b* expression domains appears to be unique to grasses and suggests an important functional distinction between these two proteins. However, functional analysis of *PIN1a* and *PIN1b* in Brachypodium has not cleanly delineated what these functions are; while single mutants do have subtle phenotypes, double mutants have much clearer phenotypes, suggesting that to some extent *PIN1a* and *PIN1b* are redundant, rather than subfunctionalized. More detailed work is therefore needed to understand the exact roles these proteins play in grass shoot meristem function.

Functional studies have identified further PIN involvement in controlling tillering, a defining aspect of grass shoot architecture and a key determinant of crop yield, especially in rice. Rice has two PIN1 homologues (Li *et al.*, 2019) (*OsPIN1a* and *OsPIN1b*; the genes *OsPIN1c* and *OsPIN1d* are actually PIN11 clade members), Whilst the phenotypic effects of single *ospin1a* and *ospin1b* knockouts were minor, double mutants have drastically reduced plant height and increased tillering (Xu *et al.*, 2005). Expression of the *PIN11* genes *OsPIN1c* and *OsPIN1d* was found to be lower than that of the *PIN1* homologues in both the shoot and root, but still at relevant levels in the meristem (Wang *et al.*, 2009). *ospin1c* and *ospin1d* single mutants still showed some decrease in plant height and tiller number, though the double mutant of these two genes was no more extreme than either single mutant (Li *et al.*, 2019). However, this double mutant line did produce plants with no panicle and lacking secondary branches and spikelets, analogous to *sopin1* mutants in Brachypodium. Further investigation into the roles of specific *PIN1* and *PIN11*/*SoPIN1* genes may reveal the existence of subfunctionalization, and the possibility of such phenomena to have driven structural differentiation between the *Poaceae*.

In wheat, *TaPIN1-6* represents a complex of six genes with one homeologue in both the A and D genomes (*TaPIN1-6a* and *TaPIN1-6d*) and four in the B genome (*TaPIN1-6b1–TaPIN1-6b4*). Meanwhile *TaPIN1-7* has a single homeologue in each genome (Yao *et al.*, 2021). Expression analyses of these genes showed that they are highly expressed, particularly in the stem apex and axillary buds. RNAi-based disruption of *TaPIN1-6* and *TaPIN1-7* function resulted in transgenic wheat lines that produced significantly more tillers than the wild type. Additionally, the knockdown lines also produced more ears, almost certainly as a direct function of the increased tiller number (Yao *et al.*, 2021). However, the ears of these lines also produced fewer spikelets per ear, fewer grains per ear, and (in two of the three RNAi lines) reduced thousand-grain weight. Interestingly, these reductions did not completely negate the positive effect of the increased ear number, and the transgenic lines all produced a significantly increased yield over the wild type. This result is contrary to the typical result of increased ear/reduced seed lines in wheat, which tend to exhibit a deceased yield, and hence the proposal of a wheat ideotype with a very low number of highly productive ears (L. Chen *et al.*, 2020). Here, the study of auxin transport in grasses not only indicates a role in shoot architecture for PINs but highlights the potential value of such knowledge in developing novel lines for higher yield agriculture.

Intriguingly, functional analysis suggests that PIN9 proteins might also play a distinct, and presumably novel, role in regulation of grass shoot architecture. Early expression analysis found *OsPIN9* to be particularly highly expressed in the root and stem base (Wang *et al.*, 2009), and further study more specifically located this high expression to the vascular tissue of shoot junctions and in tiller buds (Hou *et al.*, 2021). *OsPIN9* is up-regulated by cytokinin (Wang and Li, 2005) and by ammonium (Hou *et al.*, 2021). As a result of its induction by ammonium, *OsPIN9* was investigated as a candidate gene involved in the increased tillering displayed by rice in high ammonium conditions, such as flooded paddy fields. Tiller number was reduced in *ospin9* mutant lines compared with the wild type in paddy conditions, and tiller number was increased in overexpression lines (Hou *et al.*, 2021). Analysis of these overexpression lines when grown with only ammonium as a nitrogen source identified an increased rate of tiller bud outgrowth as the source of the increased tillering. Unusually for a non-canonical PIN protein (which are typically localized in the endoplasmic reticulum), it was shown that OsPIN9 is plasma membrane localized *in vivo*, with evidence of the capacity for OsPIN9 to influence IAA distribution. Similar to *OsPIN9*, *ZmPIN9* in maize exhibits no expression in the tassel or ears, and instead appears to be expressed solely in the roots and nodes (Forestan and Varotto, 2012), although no functional data are available for this protein. Taken together, this work therefore provides evidence of a grass-specific PIN protein playing a novel role in auxin transport in nodes, with functional consequences for a key aspect of grass shoot architecture.

No studies into a functional role for PIN10 proteins in grass shoot architecture currently exist, but expression data are tantalizing with regard to a divergent function from PIN3-like proteins in other species, and a possible role in specialized grass architecture. *ZmPIN10a* and *ZmPIN10b* are specifically expressed in maize inflorescences, with *ZmPIN10a* expression appearing to be greater in the male inflorescence than in that of the female, while the expression of *ZmPIN10b* was found to be less than of *ZmPIN10a* in all samples from both inflorescences, except for 3 mm along the female inflorescence (Forestan and Varotto, 2012). Similar expression patterns have been observed for *OsPIN10a* and *OsPIN10b* in rice. As in maize, *OsPIN10a* appears to be more highly expressed than *OsPIN10b* in most tissues, including the stem, stem base, and young panicle; however, *OsPIN10b* is expressed more highly in the vein, hull, and anther of developing floret organs (Wang *et al.*, 2009). These results suggest that further investigation regarding PIN10 proteins as specific regulators of inflorescence development and function is warranted.

The case of PIN8 in grasses also seems intriguing, at least as far as expression studies suggest. For instance, *ZmPIN8* is very highly expressed in the young and developing seeds of maize, whilst *OsPIN8* shows very little expression in these structures (Matthes *et al.*, 2019). Additionally, whilst the *PIN8* genes of both species are highly expressed in the immature inflorescence, *OsPIN8* appears to then be down-regulated in expression in the mature inflorescence, where *ZmPIN8* is still expressed at a similar level to earlier in development. Meanwhile Arabidopsis *PIN8* exhibits a completely different expression profile, showing very low expression in the seeds and inflorescence, but being highly expressed in the stamens, where these two grass *PIN8* genes are expressed at extremely low levels. Such diversification in expression may therefore indicate functional diversification between the *Poaceae* and other angiosperms, and even between members of the *Poaceae*.

PIN-mediated auxin transport is finely regulated not only by expression of the PIN proteins themselves, but also through post-translational modifications, and trafficking and localization to particular areas of membranes. In Arabidopsis, the PINOID-family of serine/threonine kinases phosphorylate PIN proteins in the loop domain to modulate their subcellular localization (Christensen *et al.*, 2000; Benjamins *et al.*, 2001; Friml, 2003). In maize, the PINOID homologue BARREN INFLORESCENCE2 (BIF2) functions similarly (Wu and McSteen, 2007), phosphorylating ZmPIN1a, and thus controlling its cellular localization in developing inflorescence meristems in normal ears and tassels (Skirpan *et al.*, 2009). *pinoid* (*pid*) mutants have previously been shown to exhibit a similar developmental phenotype to *pin* mutants, namely a defect in floral meristem initiation resulting in a pin-like inflorescence (Bennett *et al.*, 1995; Christensen *et al.*, 2000; Benjamins *et al.*, 2001). This developmental defect is also observed in maize *bif2* mutants which fail to initiate spikelet pair meristems (Barazesh *et al*., 2009). Other work showed that *bif2* maize mutants lacked a compact group of PIN-expressing cells in the tassel and ear, and had altered *ZmPIN1a* and *ZmPIN1b* expression patterns (Carraro *et al.*, 2006). Intriguingly, allelic variation of *bif2* results in variation in maize tassel architecture via the modulation of auxin transport during vegetative and inflorescence meristem development (Pressoir *et al.*, 2009). This finding is particularly salient for the core hypothesis of this review as it presents a clear instance of genetic changes in auxin biology resulting in developmental changes in grass shoot architecture. The orthologous rice protein OsPINOID (OsPID) also regulates shoot architecture through the regulation of auxin transport, interacting at least with OsPIN1a and OsPIN1b to do this (Wu *et al.*, 2020). Unlike *bif2*, *Ospid* mutants have no defect in the initiation of spikelets, but have abnormal stigma, style, and ovule development in flowers (He *et al.*, 2019; Xu *et al.*, 2019). Thus, despite their close relationship, *ZmBIF2* and *OsPID* have distinctly different roles in the control of shoot architecture, suggesting that changes in *PID* expression during grass evolution could contribute to differences in shoot architecture between the grasses.

Our understanding of PIN function in shoot architecture development is built on phenotypic observations of a variety of *pin* mutants, coupled with observation of PIN protein localization in tissues. A current lack of *pin* mutants in grasses other than maize and rice is a major roadblock to improving understanding of grass-specific PIN function and regulation of shoot architecture. However, the apparent innovations in both the expression and function of PIN proteins in grasses certainly warrant further investigation for potential roles in grass-specific developmental innovations.

## Nuclear auxin signalling

By far the best understood auxin signal transduction pathway in plants is the nuclear TIR1/AFB pathway, in which Aux/IAA-family transcriptional repressors are targeted for degradation by the action of an SCF–E3-ubiquitin ligase complex containing TIR1/AFB F-Box proteins, in an auxin-dependent manner (Hagen, 2015; Salehin *et al*., 2015). The degradation of the Aux/IAA proteins releases ARF (Auxin Response Factor) transcription factors to modulate expression of genes with promoters containing Auxin Response Elements (AuxREs or AREs). Recent work has shown that multiple other auxin signalling pathways exist (Ang and Østergaard, 2023), but here we will focus on canonical, nuclear auxin signalling since it is the most functionally important and has therefore been subject to the most (and indeed only) study in grasses. Multiple components of this signalling pathway, such as the ARFs, AUX/IAAs, and TIR1/AFBs, exist in multiple copies within each grass species (Table 1), often varying in expression profile and sequence, and are therefore potentially a likely source of significant interclade and interspecies variability.

ARFs are categorized into three conserved clades (A, B, and C), (Finet *et al.*, 2013; Flores-Sandoval *et al*., 2015; Galli *et al.*, 2015, 2018), wherein clade A ARFs act as transcriptional activators, clade B act as transcriptional repressors, and clade C may have no direct function in auxin signalling (Flores-Sandoval *et al*., 2018). Aux/IAAs are categorized into nine clades, two of which are thought to be monocot specific (Matthes *et al.*, 2019). TIR1/AFBs are categorized into three subclades, which pre-date monocot–dicot divergence. Although these core clades are shared between species and monocots and dicots, differences in copy number exist from species to species (Table 1) and differences in expression and sequence exist between paralogues.

In Arabidopsis, single knockouts of TIR1/AFBs, ARFs, and Aux/IAAs typically result in a mild phenotypic response, but multiple knockouts result in plants with multiple severe defects in auxin-mediated development or that often fail to germinate entirely (Dharmasiri *et al.*, 2005; Sakamoto *et al.*, 2013; Prigge *et al.*, 2020; Uzair *et al.*, 2021). Expression studies in rice and maize show that many of the TIR1/AFB genes in these species exhibit similar expression profiles to the other TIR1/AFBs within the same species but vary between species (Matthes *et al.*, 2019). For instance, almost all maize TIR1/AFB genes are highly expressed in young seeds and the inflorescences, whereas all five rice TIR1/AFBs show relatively low expression in the seeds and most show low expression in the inflorescences. These data could suggest a diversity in function between orthologues (e.g. *ZmAFB4/5B2* and *OsAFB4/5B*) and between paralogues in the same genome (e.g. *OsAFB2/3A* show a much higher expression level in mature inflorescences than the other rice TIR1/AFB genes). In terms of functional analyses, mutation of rice *TIR1/AFB* genes showed that single mutant lines of *Ostir1* and *Osafb2* produced shorter plants, more tillers, and fewer grains per panicle (Guo *et al.*, 2021). *Ostir1 Osafb2* double mutants exhibited even more severe differences in height, tillering, and grain number compared with the wild type and had a significantly reduced grain size and mass. This is similar to Arabidopsis, where TIR1/AFB single and higher order mutant lines (including *tir1* and *afb2* mutations) also exhibit reduced height, increased branching, and reduced seed number (Prigge *et al.*, 2020). Generation of higher order TIR1/AFB mutants in maize and rice is a key goal for expanding this knowledge into grasses (Galli *et al.*, 2015; Qiao *et al.*, 2018).

These observations of diversity between paralogues also appear to hold true for Aux/IAAs and ARFs, and, given the increased number of family members of these proteins, diversity in location and timing of expression is even greater than in TIR1/AFBs. For instance, *ZmIAA17* shows very low expression levels in the embryo, whereas the closely related *OsIAA9* is very highly expressed. Meanwhile, *OsIAA20*, which is classified within the same clade as *OsIAA9*, shows very little expression in the embryo (Matthes *et al.*, 2019). One of many possible examples for the ARFs is the high expression of *ZmARF1* in the developing seed, compared with the low expression of closely related genes *OsARF21* and *ZmARF27* (Matthes *et al.*, 2019). However, it should be noted that the expression profiles compared in this study are from multiple experiments performed by different groups without a unified control. Dedicated experiments directly comparing potential differences between these orthologous genes must be performed to confirm the existence of these differences.

Functional analysis of Aux/IAAs in grasses is generally lacking. In Arabidopsis, Aux/IAAs are highly redundant, and only semi-dominant mutations in single genes typically produce phenotypes. The situation seems similar in grasses, with few reported Aux/IAA mutations affecting shoot architecture. However, there are some striking counter-examples. In maize, semi-dominant mutants in *ZmIAA27* (*Barren Inflorescence1*) and *ZmIAA20* (*Barren Inflorescence4*) strongly affect shoot architecture, producing tassels with a reduced number of branches and spikelets, and ears with reduced length and kernel number (Galli *et al.*, 2015). Double mutants show more extreme versions of these phenotypes, with *pin*-like inflorescences as a result of severely impaired axillary shoot meristem initiation. Interestingly, single knockout mutations of OsIAA23 produce strong shoot architectural phenotypes, including dwarfing and reduced tillering (Jiang *et al*., 2019), but few other rice Aux/IAAs have been found to produce phenotypes.

Compared with Aux/IAAs, there is much more evidence for ARFs as regulators of shoot architecture. In rice, expression profiling of *ARF* genes shows that relatively few are highly expressed in the shoot but, of the three clade A *OsARF* genes with this expression profile, all are functional regulators of rice shoot architecture (*OsARF6*, *OsARF17*, and *OsARF19*) (Matthes *et al.*, 2019). Overexpression of *OsARF19* results in reduced height (Zhang *et al*., 2015), while RNAi lines and *osarf19* null mutants both exhibit disrupted floral organ development, producing abnormal florets (Zhang *et al*., 2016), suggesting that *OsARF19* is a key regulator of rice shoot architecture, further supported by its expression profiles in young panicles. Along with *OsARF12* and *OsARF25*, *OsARF6* and *OsARF17* are targeted for repression by miR167, and overexpression of this miRNA causes reduced stature and reduced tillering (Liu *et al.*, 2012). Double knockout mutants in *OsARF6* and *OsARF17* cause an increased flag leaf angle (Huang *et al.*, 2021), and OsARF12, OsARF19, and OsARF25 also regulate flag leaf angle (Li *et al.*, 2020). Three clade B ARF genes (*OsARF7*, *9*, and *15*) and one clade C ARF gene (*OsARF18*) in rice also show high shoot expression. All of these lack functional investigation and appear to be prime targets for further investigation of the role of auxin signalling in shoot architecture in grasses. Only two maize genes show particularly high shoot expression, *ZmARF7* and *ZmARF35* (Matthes *et al.*, 2019). The precise function of *ZmARF7* is not known; however, *ZmARF35* binds certain regions on the promoter of *BARREN STALK1* (*BA1*). *ba1* mutants exhibit reduced tillering, the same phenotype that results from the disruption of shoot-expressed rice ARF genes *OsARF6* and *OsARF17*. This link suggests that *ZmARF35* mutation would result in a similar effect and should be prioritized for functional investigation.

Other ARFs implicated in shoot architecture include *OsARF1*, where antisense expression resulted in decreased shoot height (Attia *et al.*, 2009), as did single knockout lines of *OsARF11* and *OsARF16* (Sakamoto *et al.*, 2013; Uzair *et al.*, 2021). Knockout of *OsARF11* and *OsARF16* also resulted in an increase in tillering (Sakamoto *et al.*, 2013; Uzair *et al.*, 2021). *OsARF4* (Hu *et al.*, 2018) and *OsARF25* (Z. Zhang et al., 2018) negatively regulate grain size, whereas *OsARF6* (Qiao *et al.*, 2021) and *OsARF11* (Sims *et al.*, 2021) have been implicated in the positive regulation of grain size. In wheat, expression profiling identified *TaARF4*, *9*, *12*, *15*, *17*, *21*, and *25* as potential regulators of tillering (J. Li *et al.*, 2021). Furthermore, reduced *TaARF11* expression has been identified as the causative factor in the reduced tillering of the *dwarf monoculm* (*dmc*) mutant line (He *et al.*, 2018). This interpretation of the role of *TaARF11* in tillering control is complicated by reduced IAA levels in *dmc*. The precise effects of this auxin reduction, expression of *TaARF11*, and expression of other auxin signalling genes in this mutant remain to be determined. *TaARF11* is closely related to *OsARF11* (J. Li *et al.*, 2021), but the reduced tillering that results from its disruption is in contrast to the increased tillering that results from a knockout of *OsARF11*. This example shows that changes in ARF expression could be a source of variation in shoot architecture between grass species. A picture thus emerges of ARFs playing antagonistic roles in many aspects of shoot architecture in grasses, although the currently fragmentary nature of the data makes it difficult to understand this within a holistic framework.

In addition to the synthesis, transport, and signal transduction of auxin, the diversity of downstream responses could also be a major source of diversity and functional and structural novelty in grasses. Several instances of tillering mutants have been described in this context, a perhaps unsurprising finding, considering our current knowledge of genetic control of tillering in grasses. TEOSINTE BRANCHED 1 (TB1) was first identified as a central coordinator of tillering in maize, and orthologous proteins have been discovered with equivalent function in wheat (TaTB1) (Dixon *et al.*, 2018), rice (OsFC1) (Takeda *et al.*, 2003), sorghum (SbTB1) (Kebrom *et al*., 2010), and barley (HvINT-C or HvVrs5) (Ramsay *et al.*, 2011; Zwirek *et al*., 2019). These genes act to repress axillary meristem outgrowth, and their expression is a known downstream target of auxin signalling (del Rosario Cárdenas-Aquino *et al*., 2022). Although broadly similar, the phenotypic results of altered expression of these genes are not directly equivalent, suggesting that evolutionary changes to this central coordinator could underpin some of the variation in grass shoot architecture between species.

Another relevant example is HIGH-TILLERING DWARF1 (HTD1) in rice. HTD1 is an essential enzyme in the synthesis of strigolactone, a molecule which has its own signalling capability and influence on shoot architecture (Jiang *et al.*, 2013; Bürger and Chory, 2020; Mashiguchi *et al*., 2021). *HTD1* expression is induced by auxin and is a widely conserved regulator of shoot architecture (Zou *et al.*, 2006). An example in maize is the *BARREN STALK* (*BA*) genes. BA1 (Gallavotti *et al.*, 2004) and BA2 (Yao *et al.*, 2019) both influence axillary shoot meristem formation, and their mutants exhibit disruption to ear and tassel development, with reduced branching and spikelet number. BA1 functions downstream of auxin signalling and has been proposed to interact with BA2 to regulate proper axillary shoot meristem development, as a consequence of auxin establishing normal phyllotaxic patterning in the inflorescences (Yao *et al.*, 2019).

From current research, a significant body of evidence suggests that (unsurprisingly) genes involved in auxin signalling influence the development of shoot architecture in grasses, and that differences in sequence, location of expression, and timing of expression exist between these genes. Taken together, these further build the case for such changes potentially underlying changes in shoot architecture between members of the *Poaceae*. Future investigation into sequence and expression differences between signalling elements in grasses, the generation of higher order mutants, and the identification of functional similarities and differences between paralogues will test whether there is a link between innovation in auxin signalling and innovation in grass shoot architecture.

## Conclusions

So how does the most important hormone make the most important plants? In this review, we have attempted to ask whether innovations in auxin biology are associated with the morphological innovations in the shoot architecture of the grasses. There is currently no clear answer to this question, but there are tantalizing hints that it may be the case. The dramatic changes in PIN protein structure and complement between the grasses and their near relatives in the Poales implies a strong selective drive for novel functionalities, given that these altered structures are then highly conserved within the grasses. The expression of these genes, and the functional evidence where available, certainly implicates them in the development of shoot architecture, but it is too early to firmly associate specific changes in PIN proteins with specific morphological effects. The existence of apparent grass-specific clades of auxin biosynthesis and signalling components, and the changes in expression and function relative to known paralogues in Arabidopsis, does indicate significant auxin innovation between the grasses relative to other plant species. There are also clear examples, such as the functional differences between the orthologues VT2/FB/TaTAR2.1, that support the idea that changes in expression level or timing of auxin synthesis genes might be associated with differences in shoot architecture within the grasses.

However, as we have also outlined, there remains a lack of detailed understanding of auxin biology in the ‘big three’ cereals (rice, maize, and wheat), and little species-specific knowledge exists for other, still significant cereals, such as barley, sorghum, and millets. Future work would be best focused on the functional analysis of pathways that identify and connect changes in gene sequence, associated changes in auxin distribution and response, and causally connected changes in shoot architecture. Many studies have been discussed in this review that have produced a plethora of expression data, but such knowledge remains of limited use without associated investigation into the phenotypic effects of such differences, and the underlying presence and activity of auxin. Additionally, the systematic generation and study of single and multiple mutants for auxin-related families in key grasses, based on current knowledge from Arabidopsis, would be a rational next step. The rapid advances in the ability to perform multigene CRISPR in grass species, allowing functional redundancy to be overcome even in species with complex genomes, should make such an approach more feasible.

Overall, understanding the basis of the morphological innovations in the grasses, whether these are driven by changes in auxin biology, or changes in other developmental pathways, would be hugely beneficial in determining the possibility and mechanisms for the continued improvement of grass shoot architecture, with implications for improved food security and feeding the increasing global population with reduced arable land.
